# SWATH-MS-facilitated proteomic profiling of fruit skin between Fuji apple and a red skin bud sport mutant

**DOI:** 10.1186/s12870-019-2018-1

**Published:** 2019-10-24

**Authors:** Mo-Xian Chen, Chao Sun, Kai-Lu Zhang, Yu-Chen Song, Yuan Tian, Xi Chen, Ying-Gao Liu, Neng-Hui Ye, Jianhua Zhang, Shenchun Qu, Fu-Yuan Zhu

**Affiliations:** 1 Co-Innovation Center for Sustainable Forestry in Southern China, College of Biology and the Environment, Nanjing Forestry University, Nanjing, 210037 Jiangsu Province China; 2 College of Horticulture, Nanjing Agricultural University, No. 1 Weigang, Nanjing, 8210095 China; 3 Medical Research Institute, Wuhan University and SpecAlly Life Technology Co., Ltd, Wuhan, China; 4 State Key Laboratory of Crop Biology, College of Life Science, Shandong Agricultural University, Taian, Shandong China; 5 Southern Regional Collaborative Innovation Center for Grain and Oil Crops in China, Hunan Agricultural University, Changsha, 410128 China; 6 Department of Biology, Hong Kong Baptist University, and State Key Laboratory of Agrobiotechnology, The Chinese University of Hong Kong, Shatin, Hong Kong

**Keywords:** Bud sport, Skin color, SWATH-MS, Post-transcriptional regulation

## Abstract

**Background:**

Apple is one of the most popular fruit crops world-wide and its skin color is an important quality consideration essential for commercial value. However, the strategy on genetic breeding for red skin apple and the genetic basis of skin color differentiation is very limited and still largely unknown.

**Results:**

Here, we reported a bud sport mutant of Fuji apple with red skin color and enhanced anthocyanins accumulation. Quantitative SWATH-MS (sequential window acquisition of all theoretical spectra-mass spectrometry) proteomics investigations revealed proteome changes in the apple red skin bud mutation and a total of 451 differentially expressed proteins were identified in apple skin. The mutant showed significantly increased expression levels of photosynthesis-related proteins, stress-related proteins as well as anthocyanins biosynthesis pathway. On the other hand, substantial downregulation of mitogen-activated protein kinase 4 (MAPK4) and mevalonate kinase (MVK) were detected, indicating a promising role for the red skin color development in the mutant. Furthermore, we also hypothesize that a post-transcriptional regulation of the skin color formation occurs in the mutant through the advanced SWATH-MS analysis.

**Conclusion:**

Our work provides important information on the application of proteomic methods for analysing proteomes changes in Fuji apple and highlights a clade of regulatory proteins potentially contributing for the molecular breeding of fruit skin color.

**Supplementary material:**

**Supplementary information** accompanies this paper at 10.1186/s12870-019-2018-1.

## Background

Apple (*Malus domestica*) is one of the most economically important fruit crops world-wide and its demand from consumers continuously grow based on the nutritional value and broad health benefits. Apple quality mainly depends on multiple characteristics including size, taste, sweetness, texture and bright skin-color [1, 2]. Interestingly, we often use red skin-color as phenotypic outward assessment for judging the quality of apples. Actually, optimize any one of them would bring quality improvement in apple cultivation.

Apple fruit coloration is considered as an apparent indicator of maturity and outer quality during the fruit breeding [3–5]. Basically, a group of natural flavonoid compounds especially anthocyanins are essential for fruit coloration, accounting for 80% of total skin pigmentation in apple [6]. And most of genes including phenylalanine ammonia-lyase (PAL), chalcone isomerase (CHI), dihydroflavonol4-reductase (DFR) involving the anthocyanins biosynthetic pathway have been also well characterized in apple [7–9]. The anthocyanins accumulated in the red skin have the antioxidative and hepatoprotective effects contributing to the health benefits of fruits [10, 11]. However, besides anthocyanins, various unknown genetic and environmental factors have markedly influenced on the skin colour of apple. Few studies have been carried out to investigate the underlying molecular components on the comparisons between different cultivars and mutation. The Asian apple cultivar “Fuji” was commercialized in China whereas a red bud mutation of “Fuji” apple showing red skin pigmentation was recently discovered in Jiangsu Province, China. This bud mutation apple is always red spanning the entire skin-coloration development stage with stable phenotype through continuous observation and grafting experiment. Therefore, to dissect the potential molecular mechanisms of skin-coloration from this mutant and identify the candidate genes controlling the formation of red color is very meaningful for the molecular breeding of apple cultivation.

Transcriptomic and metabolomics analyses on apple fruit color have been investigated to provide insights on the global mRNA profiling and pigmentation metabolites in the formation of apple skin color [6, 12]. However, genes expression regulation may not adequately reflect the corresponding protein expression due to the various posttranscriptional modifications [13]. So far, understanding on the regulatory mechanisms of apple fruit coloration and mutation at proteome levels remains unclear due to the few proteomic studies reported. In this study, changes in the proteome profile between the “Fuji” apple and a red bud mutation were determined by the SWATH-MS (sequential windowed acquisition of all theoretical mass spectra) approach which has been extensively applied into research model plant such as Arabidopsis and rice [14, 15]. SWATH-MS experiment perform on the data-independent acquistion (DIA) mode, which combines the advantages of high-throughput of shotgun proteomics and high reproducibility of SRM proteomics, leading to the large-scale protein identification and protein quantification accuracy [16]. It has received considerable attention in plant proteome research due to the higher accuracy and reproducibility in comparison to the conventional approach such as 2D-gel (Two-dimensional gel electrophoresis) and iTRAQ. In present work, 1470 unique proteins were quantitatively identified in “Fuji” apple skin. Amongst them, a total of 451 differentially expressed proteins were observed during the fruit development between “Fuji” apple and its bud mutation. Go and pathway enrichment analysis revealed substantial proteome changes distributing on these biological aspects including photosynthesis and energy metabolism, stress related proteins as well as anthocyanins biosynthesis pathway. Following bioinformatics data analysis would offer considerable information on proteome differentiates between the “Fuji” apple and its bud mutation, acquiring comprehensive understanding on the molecular mechanisms of skin-color development in apple.

Taken together, our SWATH-MS based proteomic investigation may reveal their genetic differences of apple skin-color formation at the protein levels and determine crucial modulator for potential application to the high quality and large fruit-shaped as well as bright color apple cultivars in future.

## Result

### SWATH-MS quantitative proteomics analysis in the Fuji red skin color mutant (Fuji_M)

**Fig. 1:**
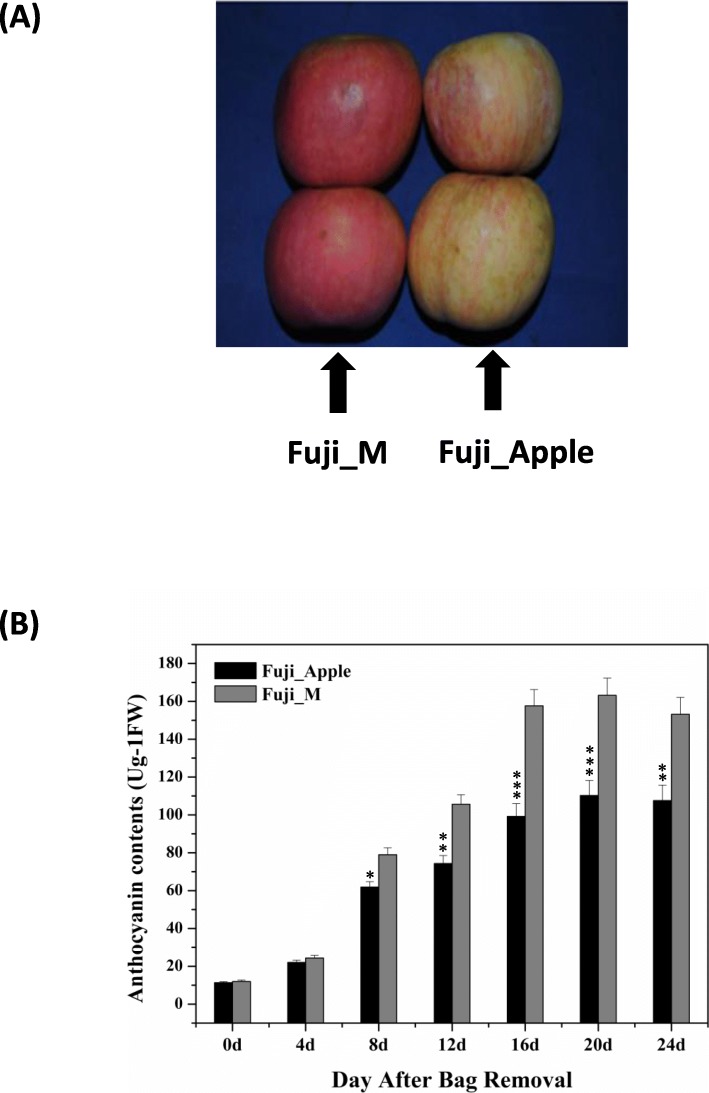
Phenotypes of “Fuji” apple and its red bud mutation (Fuji_M). **a**. Representative phenotype was photographed at 16 day after bag removal. **b**. Anthocyanin contents measurement for sampling. Data are mean of five replicates per treatment. * *P*<0.05, **P<0.01,***P<0.001 by Student’s t test

**Fig. 2:**
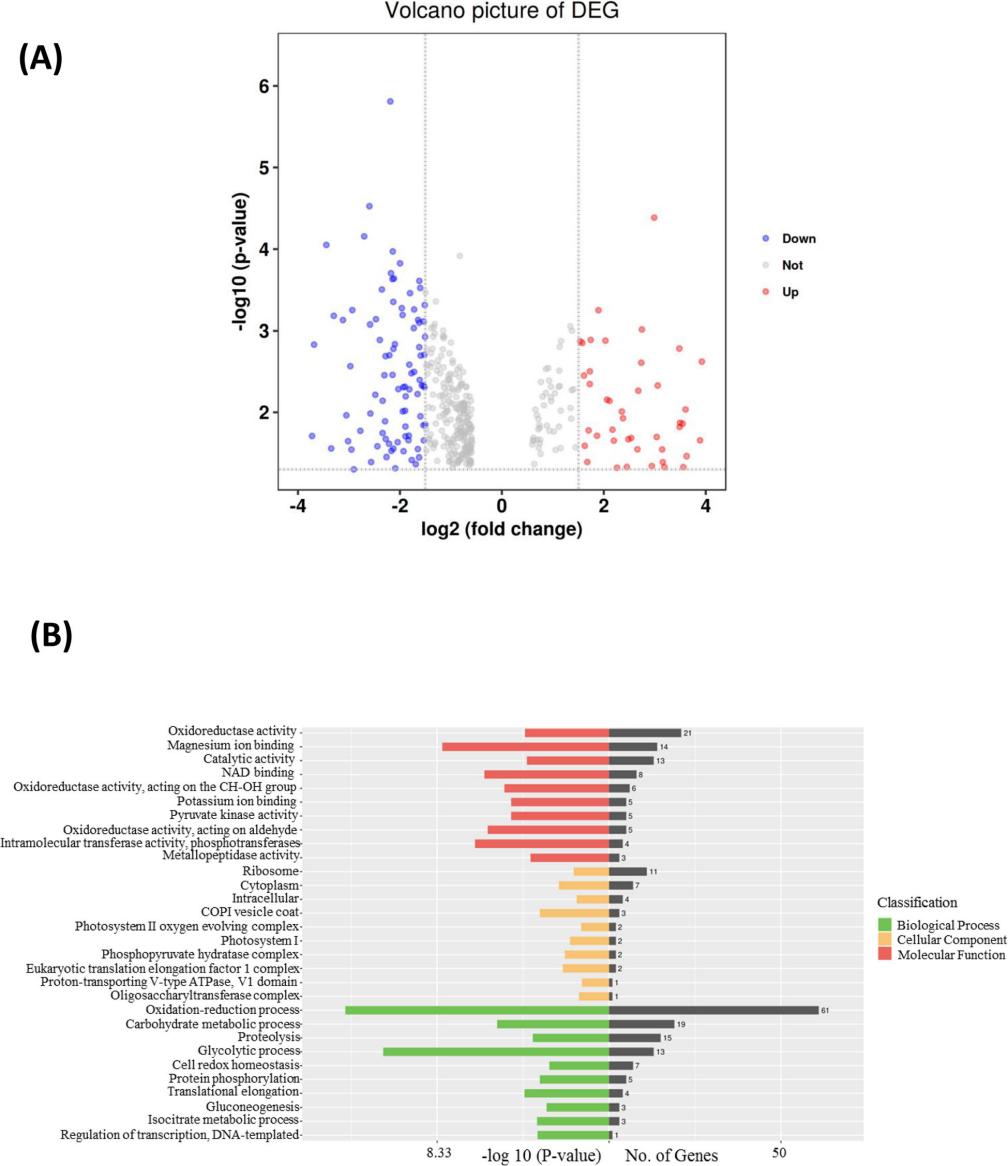
Functional categorization of the differentially expressed proteins by the Gene Ontology (GO) analysis. **a** The volcano picture showing the distribution of the differentially expressed proteins with different fold changes. **b** The plots reveal the differentially expressed proteins distribution based on three ontologies including Biological Process, Cellular Component and Molecular Function

Fuji apple (*Malus x domestica* Brokh cv. Fuji) and the red skin color mutant Fuji_M were found in Shilaojia, Jiangsu Province during the harvest season of 2017. Their young fruits were grown for 30 days after pollination and then were covered with brown bags. The differentiate color of fruits were photographed at 16 day after bag removal as shown in Fig. 1a. It was well known anthocyanins have been identified as an important indicator responsible for fruit skin color formation in many plants [3, 4]. Therefore, to ensure an appropriate sampling time point between Fuji_apple and Fuji_M for proteomic analyses, the endogenous anthocyanin contents were determined by LC-MS/MS. After bag removal, the anthocyanin levels increased gradually during the fruit maturation but the accelerated rate in the Fuji_M were higher than Fuji_apple (Fig. 1b). The maximum differences of anthocyanin contents occurred at 16 days after bag removal for Fuji_apple and Fuji_M (Fig. 1b). Therefore, the 16 DABR (fruits were exposed to normal sunshine after bag removed) were chosen for this proteome study to investigate the proteome changes between Fuji_apple and Fuji_M during the fruit maturation by the SWATH-MS analysis. After merging the data from six biological replicates, approximately 1470 unique proteins were identified and quantified with critical false discovery rate of 1% (Additional file 1: Table S1). Proteins with a fold change of above 1.5 or below 0.67 (*P* < 0.05) were considered as differentially expressed proteins (DEPs) in this study. Accordingly, in all of 451 proteins showing significant protein abundance changes between Fuji_apple and Fuji_M with 135 increased and 316 decreased (Table 1). A volcano picture of differentially expressed proteins also revealed numbers of DEPs showing increased abundances in red skin color mutant were 2–3 folds than in the Fuji_apple (Fig. 2a), indicating some crucial regulators or pathways were induced potentially contributing for the red skin phenotype.

**Table 1:** Distribution of DEPs numbers

CK VS M	Number of unique proteins
Increased (FC ≥1.5, *P* < 0.05)	135
Decreased (FC ≤ 0.67, P < 0.05)	316
Total	451

**Fig. 3:**
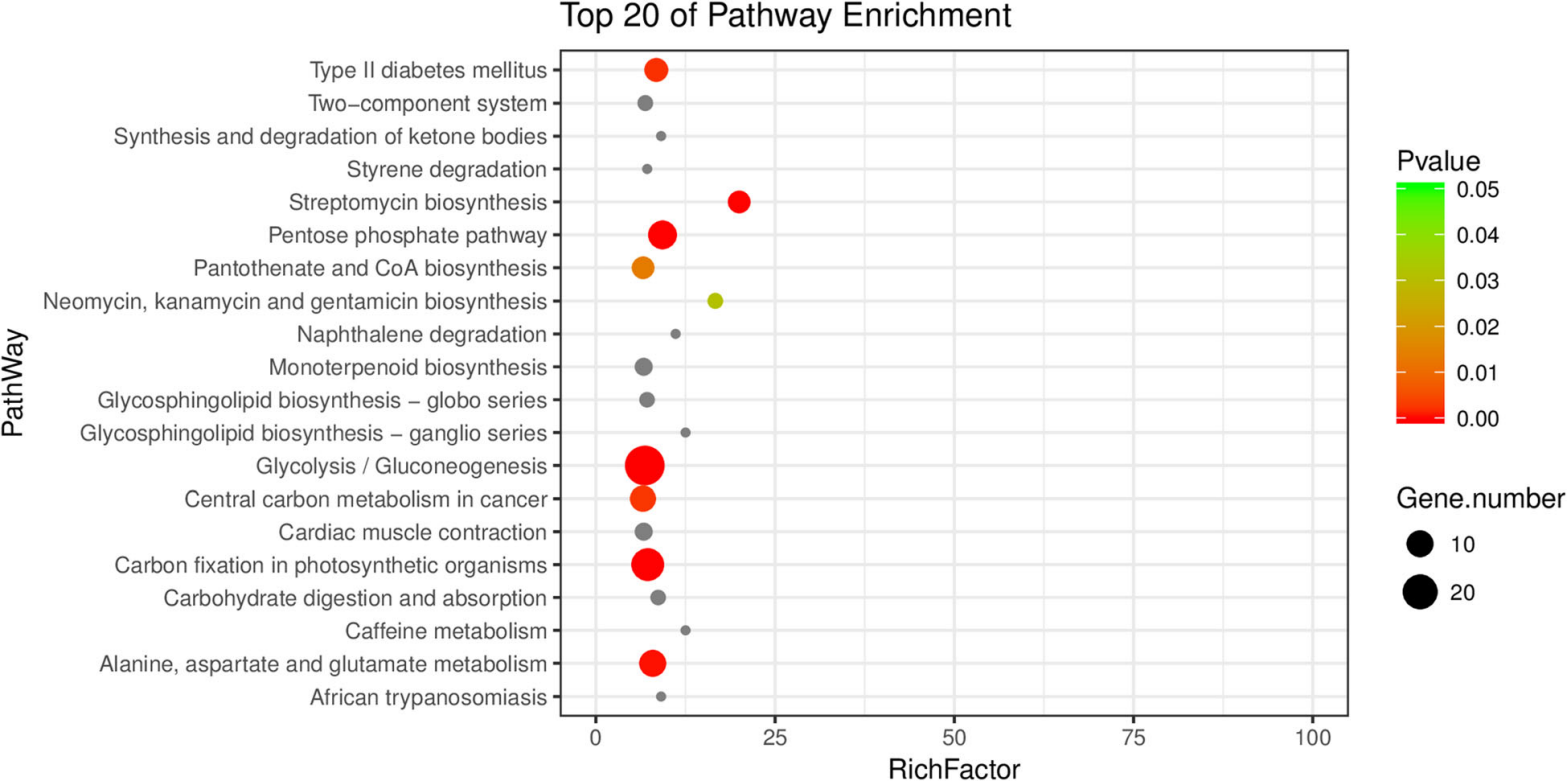
KEGG classifications on the DEPs. RichFactor represents the degree of enrichment

Subsequently, functional classification of those DEPs was performed by the Gene Ontology (GO) analysis including Biological Process, Cellular Component and Molecular Function. Accordingly, proteins belonging to the categories “Oxidation-reduction process” and “Carbohydrate metabolic process” contain the largest number of DEPs, indicating that these courses were greatly affected in the red skin bud mutation (Fig. 2b). It is also worth noting that proteins possessing oxidoreductase activity and magnesium activity were highly represented in the GO term of “Molecular Function”, indicating these enzymes were co-ordinately regulated for the red skin color formation. The majority of the DEPs assigned with the GO term of “Cellular Component” were distributed in ribosome and cytoplasm (Fig. 2b). Therefore, these findings from GO analysis indicated that different corresponding biological processes or enzymes were activated or repressed in red skin bud mutation to fulfil blushed properties in apple. Moreover, KEGG (Kyoto Encyclopedia of Genes and Genomes) pathway classifications were also conducted on the DEPs offering a quick view to the most significant pathway in the bud mutation (Fig. 3). We listed top 20 of pathway such as glycolysis, carbon fixation in photosynthetic process, amino acid metabolism upon the mapping of DEPs (Fig. 3), among which should be the key for the differentiate phenotype in apple.

### Photosynthetic and energy metabolism proteins

The extent of photosynthesis is critical for the fruit skin color changes. The fruit color from green to red always depend on the reduction or suppression of chlorophyll content to appear the color of anthocyanins by the regulation of photosynthesis proteins [17, 18]. In this study, there are 18 photosynthesis-related proteins identified with altered abundances. Interestingly, most of all were up-regulated in the red skin mutant, among which are important elements of photosynthetic enzyme complexes including cytochrome b6/f, photosystem I and II (PSI and PSII) and light-harvesting chlorophyll protein complex (LHC) (Additional file 2: Table S2), strongly suggesting that the light reactions were reinforced in the red skin color mutant compared with Fuji_apple. Similarly, CO_2_ assimilation was also shown to be accelerated with increased protein abundances of Calvin cycle enzymes in the mutant, including rubiscoactivase (MDP0000944409) required for CO_2_ fixation and glyceraldehyde-3-phosphate dehydrogenase (MDP0000835914, MDP0000527995) involved in the reduction stage as well as D-ribulose-5-phosphate-3-epimerase (MDP0000276466) involving the regeneration of ribulose bisphosphate. Interestingly, NAD-dependent malate dehydrogenase (MDH) was also found to show increased abundances in the mutant, which is a key enzyme catalysing hydrogen ion of hydroxyl in malice acid for oxaloacetate regeneration, indicating the enhancement of respiratory metabolism in the red skin bud mutation. Therefore, the overall up-regulation of photosynthetic activities in this mutant probably due to the demand of light absorption by the accumulation of anthocyanins, resulting in the improvement of the ability of light reaction, carbon assimilation as well as respiration for the red skin bud mutation.

### Upregulation of anthocyanins biosynthetic pathway in Fuji red skin color mutant

**Fig. 4:**
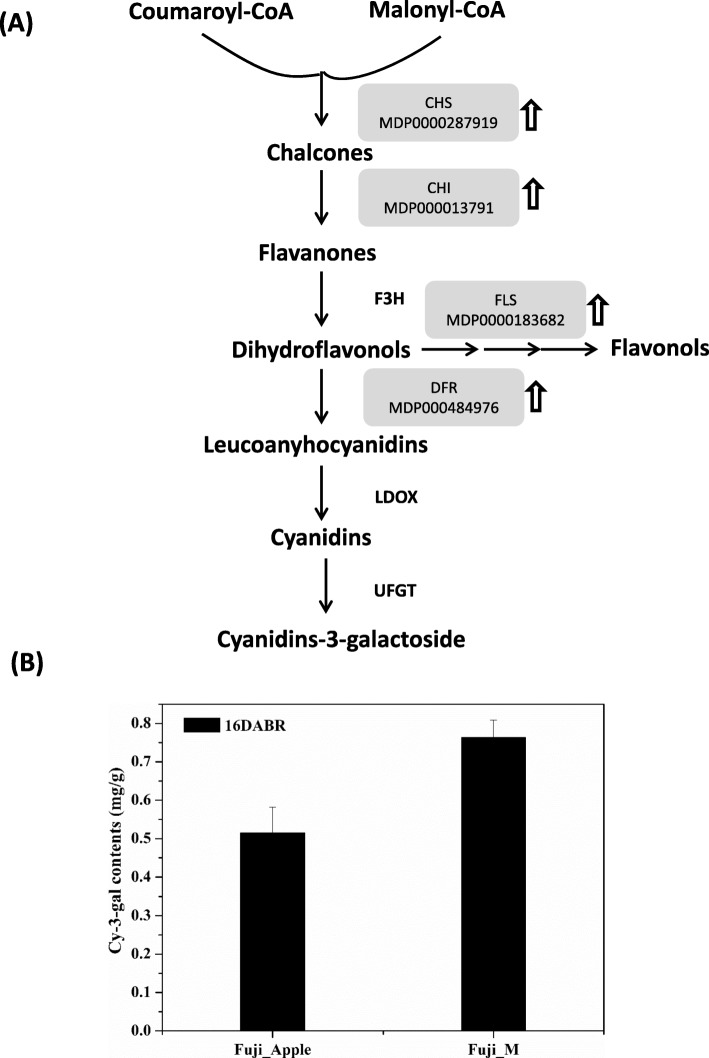
Anthocyanin biosynthesis was greatly affected in the bud mutation. **a** Several enzymes involved in anthocyanin biosynthesis showing significant protein abundances changes were mapped to the anthocyanin metabolism pathway. ⇧ indicates up-regulation. Enzyme abbreviations: CHS, chalcone synthase; CHI, chalcone isomerase; F3H, flavanone-3ß-hydroxylase; DFR, dihydroflavonol-4-reductase; FLS, flavonol synthase. **b** Cy-3-gal was detected in the Fuji_M and Fuji_Apple at 16 day after bag removal (DABR)

Anthocyanins are essential components in red coloration sources for apple skin color [5]. The red-skin cultivars accumulate more anthocyanins than the pale or white ones, which have been considered as an important fruit trait influencing the market values. Here, several key enzymes with enhanced abundance changes were participated and mapped into sequential reactions of anthocyanins biosynthesis (Fig. 4a). Anthocyanins are synthesized from coumaroyl-CoA and malonyl-CoA by the catalysation of chalcone synthase (CHS, MDP0000287919) to form chalcones. Afterward, chalcone isomerase (CHI, MDP000013791) catalyses the conversion of chalcones to flavanones. The next step is catalysed by a flavanone-3ß-hydroxylase (F3H) resulting in the formation of dihydroflavonols. On the one hand, dihydroflavonols is then transferred for reduction bydihydroflavonol-4-reductase (DFR, MDP000484976) to form leucoanyhocyanidins. On the other hand, dihydroflavonols are also entered into the production of flavonols by flavonol synthase (FLS, MDP0000183682) through sequential reactions. The conversion of leucoanyhocyanidins to cyanidins-based anthocyanins was finished by final two enzymes including LDOX and UFGT, respectively. Therefore, those clades of enzymes with increased abundances changes directly reflect the upregulation of anthocyanins biosynthesis in Fuji_M (Fig. 4a), which also consistent with results from the anthocyanins contents determination between Fuji_Apple and Fuji_M (Fig. 1b). Furthermore, we investigated the anthocyanins compositions by the LC-MS/MS analysis and observed many types of anthocyanins contained in apple skin. Particularly, the cyanidin-3-galactoside (cy-3-gal) detected in the Fuji_M was significantly increased by approximately 50% in comparison to the Fuji_Apple (Fig. 4b), which was also the most abundant anthocyanin in apple [6]. Therefore, the significant skin-color differences in Fuji_Apple and Fuji_M are probably due to the regulation of anthocyanins biosynthesis. Interestingly, this kind of regulatory mechanism is mainly based on a transcription complex, which consist of three transcription factors belonging to the MYB, bHLH and WD40 classes [4, 17], activating anthocyanin biosynthesis pathway by the MYB-bHLH-WD40 complex in most plants [19]. However, no MYB and bHLH-related proteins were found and only two WD40 repeat-like proteins (MDP0000231283 and MDP0000433881) with enhanced abundances in the mutant were quantitatively identified from this study (Additional file 2: Table S2). Several reports revealed WD-repeat proteins promotes the accumulation of anthocyanins in plants including apple, green tea and tartary buckwheat [20–22]. The apple WD40 protein regulate anthocyanin biosynthesis through the interaction of bHLH rather than the activation of MYB-bHLH-WD40 complex [23]. Therefore, the upregulation of two WD40 classes proteins caused by the disinhibition of potential mutated gene probably regulate the anthocyanins accumulation in Fuji_M, thus contributing to the red skin coloration.

### Stress related proteins

In our study, some identified differentially expressed proteins showed than increased abundances in Fuji_M were enriched in the component of stress-related proteins and they are likely to be participated in various biotic and abiotic stresses (Additional file 2: Table S2). It was well known that stress-related proteins are spontaneously synthesized for the plant resistances coping with environmental stress factors including abnormal temperature, heavy metals, drought and salinity [24, 25]. Here, several heat shock proteins (HSPs: HSP70; HSP81.4; HSP90.1; HSP101) were detected to be accumulated in the Fuji-M (Additional file 2: Table S2), which is one kind of conservative stress family proteins. For example, HSP70 is able to involving the disposal of damaged or defective proteins to proteolysis pathway through its molecular chaperone function with E2 ubiquitin ligase [26, 27]. Coincidently, we also found unique E2 (MDP0000714492) with significant increasing abundances in this red skin mutant (Fuji-M), suggesting that this kind of cellular protective machinery was conserved and essential for the red skin bud mutation (Fuji-M). Therefore, the red skin mutant probably possesses improved resistance ability compared to the Fuji_Apple, which will need further investigation. Interestingly, Hsp70 also has been reported to participate in human retinal pigment epithelium through its chaperone function with a linkage of proteolysis pathway [28], further indicating HSP70 may be involved in the apple skin pigmentation process. In addition, three S-adenosyl-L-methionine-dependent methyltransferases (SAMMTases: MDP0000277077; MDP0000268065; MDP0000776572) were also upregulated in the Fuji-M. SAMMTases basically function to transfer methyl group from the donor of S-adenosyl methionine (SAM) to various biomolecules, playing critical roles in biosynthetic pathways of major natural products including lipids, terpenoids, nucleic acids and flavonoids, particularly in their biological activities through the modulation of the methylation patterns [29]. Therefore, SAMMTases have considerable efforts on the biosynthesis of flavonoids in the apple, which are highly correlated with skin colour formation. Upregulation of SAMMTases in the Fuji_M may alter the flavonoids structural properties and flavonoids pigmentation leading to the feature of red skin.

## Discussion

### Candidate proteins responsible for the red skin mutation

Seeking target proteins for the red skin mutation in the differential expressed proteins between Fuji_Apple and Fuji_M are necessary to illuminate the molecular mechanism of this bud mutation. Here, we collected 16 candidate proteins with dramatic decreasing abundances (Fold changes > 100) in the Fuji_M as shown in Table 2. Representatively, mitogen-activated protein kinase 4 (MAPK4: MDP0000321746) exhibited 116 folds of down-regulation in the Fuji_M. MAPKs play crucial roles in the regulation of cellular signal transduction or as signalling molecules, which have been reported to participate in pigment cell development [30]. Interestingly, flavonoids have inhibitory effects on MAPK activation [31]. Therefore, disruption of MAPK4 and upregulation of flavonoids biosynthesis pathway were observed in the Fuji_M, strongly suggesting that MAPK signalling pathway probably function as a modulator in the apple skin pigmentation process. More interestingly, stress-related protein HSP70 also involved in the MAPK signalling pathway [32], which may serve as a downstream signalling molecule contributing to the red colour formation in apple. On the other hand, another candidate protein mevalonate kinase (MVK: MDP0000025413) was shown to be greatly down-regulated in the Fuji_M, which is a key enzyme in isoprenoid synthesis. Interestingly, plants use two distinct pathway including the MEP (Methylerythritol 4-phosphate) and MVA (Mevalonic acid) pathway to synthesize isoprenoid for the function in photosynthesis, pigment production and hormones biogenesis [30]. MVK belonging to the MVA pathway play as a rate-determining enzyme for the production of isoprenoid [33] and suppression on the MVK probably cause a disordered control of isoprenoid supply, which was needed for essential processes such as photosynthesis and pigmentation. Both of them are critical for the formation of skin colour in apple. Moreover, the lowest expression of candidate protein in the Fuji_M compared to the Fuji_Apple encodes a mitochondrial ATP synthase subunit delta belonging to F1 type-ATPase (MDP0000624197). It is a component of mitochondrial respiratory complexes for plants coping with stress induced oxidative damage to respiratory function [34, 35]. The severe inhibition of this protein in the mutant probably due to the huge cost of photosynthesis as an offset of the weak respiration in mitochondrial. Taken together, further genetic and molecular strategies with bioinformatics analysis will boost to confirm the roles of these candidate proteins controlling fruit skin pigmentation and validate the target protein responsible for the red skin bud mutation.

**Table 2:** Summary of the candidate proteins responsible for the red skin mutation

Protein ID	Homolog-Arabidopsis	Description	Fold change (*P* < 0.05)
MDP0000128052	AT1G56340.2	calreticulin 1a	112.7
MDP0000327254	AT1G71950.1	Proteinase inhibitor, propeptide	105.7
MDP0000211549	AT3G47590.1	alpha/beta-Hydrolases superfamily protein;	109.5
MDP0000198482	AT1G79530.1	chloroplast/plastid localized GAPDH isoforms	111.1
MDP0000202291	AT1G02560.1	nuclear encoded CLP protease 5	111.5
MDP0000321746	AT4G01370.1	MAP kinase 4	116.8
MDP0000153587	AT5G13430.1	Ubiquinol-cytochrome C reductase iron-sulfur subunit	181.4
MDP0000222212	AT5G08380.1	alpha-galactosidase 1	184.0
MDP0000147913	AT4G10490.1	2-oxoglutarate (2OG) and Fe(II)-dependent oxygenase	208.3
MDP0000812797	AT2G44350.2	Citrate synthase family protein	215.2
MDP0000523595	AT5G28060.1	Ribosomal protein S24e family protein	223.6
MDP0000182890	AT1G21880.2	lysm domain GPI-anchored protein 1 precursor	227.6
MDP0000225450	AT1G76690.1	12-oxophytodienoate reductase 2	237.7
MDP0000470429	AT1G07920.1	GTP binding Elongation factor Tu family protein	298.1
MDP0000025413	AT5G27450.2	mevalonate kinase activity	670.2
MDP0000624197	AT5G47030.1	ATPase, F1 complex, delta/epsilon subunit	1177.7

### Proteomic analysis reveals post-transcriptional changes may contribute to red skin bud mutation in apple

Several transcriptome sequencing experiments have been conducted using different plant species such as Arabidopsis, cucumber, maize and potato [36–39]. However, the protein abundance of identified differentially expressed genes remains unclear. Previous SWATH-MS based studies reported that few transcription factors have been found in the list of differentially expressed proteins [15], supporting the claims that the regulation of transcription factors largely occur in post-translational level. In present work, twelve proteins were observed to be involved in post-transcriptional regulation through RNA processing (Table 3). Referring to the splicing-related gene (SRG) summarized in SRGD database (http://www.plantgdb.org/SRGD/index.php),5 of these genes are classified into 3 major groups of splicing factors, including proteins are involved in spliceosome composition (small nuclear ribonucleoproteins, snRNP), Poly A binding and regulatory splicing factors (glycine-rich RNA binding protein) (Table 3). Amongst them, some proteins (e.g.MDP0000283985, MDP0000245145 and MDP320945) are highly homologous with RNA processing related proteins in Arabidopsis, which have been well studied to participate in splicing process such as spliceosome assembly or splicing site selection [40, 41], implying that, other than traditional transcriptional control, substantial changes in alternative splicing may exist as an extra layer of regulatory mechanism during the skin colour formation [42]. Meanwhile, MDP0000260339, a homologue of Arabidopsis glycine-rich RNA binding protein (AtGRP7) which participates in development and abiotic stress responses [43–46], was significantly up-regulated in Fuji_M. Interestingly, several isoforms of AtGRP7 proteins were also detected by proteomic analysis to respond environmental stress [47], further suggesting the post-transcriptional regulation presumably exist during apple skin color formation. In addition, a putative apple heterogeneous ribonucleoprotein (hnRNP), MDP0000614064, was found to be up-regulated in Fuji_M. Two homologous proteins (RBP45 and RBP47) has been reported previously in *N. plumbaginifolia* [48]. These two proteins have been demonstrated to be able to associate with Poly A RNA and was potential components of U1 snRNP [48]. Therefore, the putative interaction between this protein and MDP0000283985, a Poly A binding protein [49] during post-transcriptional regulation was proposed. However, the potential role of MDP0000614064 in the formation of skin color remains elusive. Besides, several RNA-related proteins for the regulation of transcription were also detected in down-regulated protein subset (Additional file 2: Table S2), indicating that complex control of RNA stability and transcriptional availability maybe important for plant skin colour development.

**Table 3:**
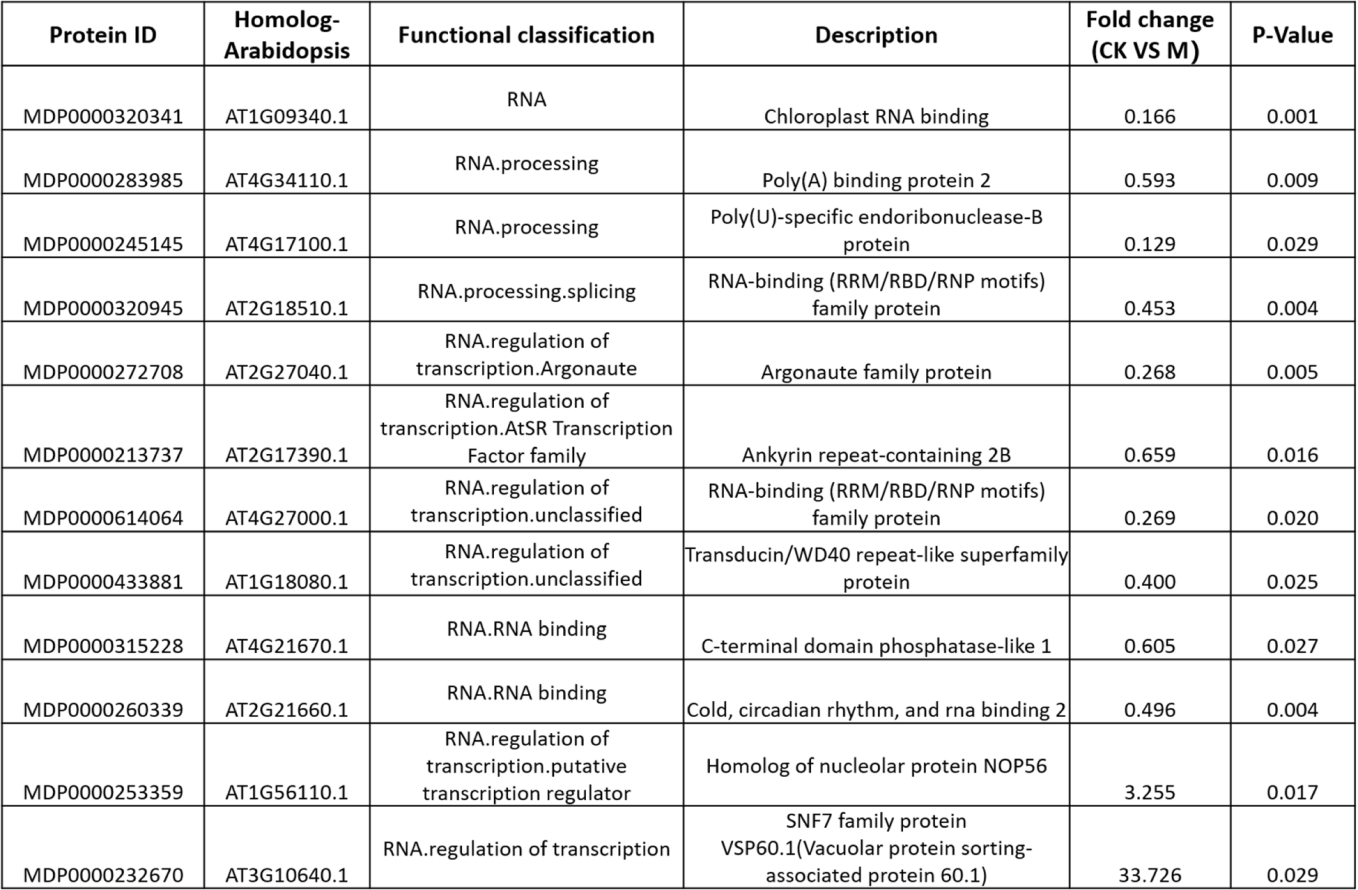
List of RNA processing related proteins in DEPs.

## Conclusion

Fruit skin colour formation seems to initiate a complex responsive network, ranging from transcriptional to post-translational control. Thus, traditional experimental design using single transcriptomic or proteomic approach will encounter more difficulties to find true regulator or pathways which are responsible for fruit skin colour. Traditional proteomic methods such as 2D-gel suffer from their instability of protein quantification and large variation [50]. Thus, as a new generation of label-free method, SWATH-MS based proteomics are believed to enhance the identification of differentially expressed proteins and improve repeatability amongst replicates at the same time [15, 51]. Besides, shot-gun proteomics are often limited by their reference databases, which can be complemented by self-constructed protein database from parallel short-read RNA sequencing [52]. The development of proteogenomics, a bioinformatic pipeline to integrate data from RNA sequencing and proteomics, become popular in recent years for both animal and plant research [42, 53]. Definitely, functional analysis such as using genetic approaches and constructing transgenic lines is strongly recommended to validate the findings from bioinformatics approach. Although several differential regulation events have been revealed by our investigation, it also possibly appears to be the effect rather than the cause for this red skin bud mutation. Therefore, the integration of proteomics, RNA-sequencing and more molecular approaches will elucidate more information in fruit skin colour formation.

The present work offered a global view to understand the molecular differentiation between Fuji apple and mutant, which revealed significant protein abundances changes in photosynthetic and energy metabolism, anthocyanin biosynthesis as well as stress responses for the differentiate colour phenotype in apple. Furthermore, several potential target proteins for the red skin mutation such as MAPK4, MVK and F1 type-ATPase were identified with greatly decreased abundances in its color mutant, among which play positively effects on the pigmentation process to the feature of red apple. Additionally, the advanced SWATH-MS analysis also revealed a certain proportion of proteins involving post-transcriptional changes serve as a regulatory role in fruit coloration. Taken together, our proteomic analysis provides new cues for further research in apple skin-color formation and will extend our understanding of skin colouration for future fruit breeding.

## Methods

### Plant materials and sampling

Bud sport are infrequent changes in phenotype particularly affecting flower and fruit color in horticultural plants such as apple and grape [54]. Here, Fuji apple (*Malus domestica* Brokh cv. Fuji) and the red skin color mutant Fuji_M from its bud sport branch were obtained from the apple orchard under standard orchard practices in city of Shilaojia, Jiangsu province, China. The Fuji apple is a commercially available cultivated variety and collected from Japan. The phenotype of this bud sport mutant has inherently appeared during the grafting and we have acquired the permission of orchard to collect samples and do further research under the national guidelines. Both flowers on normal branches and on the bud spot were pollinated. Young fruits were grown for 30 days after pollination (30 DAP) and then were covered with brown bags (size: 13 × 16 cm). Bags were removed before harvesting (150 DAP) and samples were collected for six time points: 0, 4, 8, 12, 16, 20, 24 days after bag removal (DABR) for anthocyanin contents analysis and 16 DABR was finally chosen for following proteome study. Each time point was stored and measured with six biological replicates.

### Anthocyanin contents analysis

The measurement is performed according to the previously common methods reported [55, 56]. Briefly, approximately 0.5 g of apple skin was collected and extracted by the addition of a 10 mL methanol solution with 1% hydrochloric acid in the dark at room temperature for 2 h. All samples were measured in five replicates and anthocyanins were determined by spectrophotometer at absorbance of 530 nm and 600 nm respectively. The relative anthocyanin content (Q) was calculated by this formula: Q = OD530nm-OD600nm and per 0.01 was defined as an anthocyanin content unit for convenience. Subsequently, different types of anthocyanins in apple were detected by ABI2000-QTrap mass spectrometer (Applied Biosystems). Total plant anthocyanins were extracted according to the method described above. The compositions of anthocyanins in the samples were analysed by HPLC-MS/MS using a ABI2000-QTrap and the detailed procedure was as described by [57]. Briefly, extracted and filtered samples (20 μl) were trapped on a Nucleosil 100–5 C18 column (5 μm, 150 × 2 mm, Agilent Technologies) with 95% solvent A (0.1% formic acid in water) and 5% solvent B (0.1% formic acid in acetonitrile), followed by separation in a gradient of 15–60% B over 35 min at a flow rate of 0.2 mL/min. The final LC eluted products were determined by an on-line ABI2000-QTrap mass spectrometer (Applied Biosystems) under multiple reaction monitoring (MRM) mode (positive ionization, scan range: 200–1200 m/z).

### Protein extraction and peptide preparation

Approximately 1 g of apple skin was ground into powder by liquid nitrogen in a mortar. Samples were homogenized with the pre-iced 10X vol of solution A (10% trichloroacetic acid (TCA)/ acetone) and then centrifuged at 16000 g for 5 mins. After removed the supernatants, the pellets were washed by the 10X vol of solution B (80% MeOH/0.1 M NH_4_OAc) and then centrifuged again and followed by the 10X vol of washing solution C (Chilled 80% acetone). By centrifugation with the supernatants removing, the pellets were dissolved into the 8 ml SDT buffer (4% SDS, 0.1 M DTT, 0.1 M MOPS/Cl, pH 8.0). Thereafter, dissolved sample was incubated at 95 °C for 10 mins and cooled on ice immediately for 5 mins. After centrifugation twice again, the final supernatants were obtained by 4X volume of 80% acetone for protein precipitation overnight at -20 °C. At the end, the pellets were dissolved into 1-2 ml solution buffer (6 M urea in 200 mM MOPS-Cl/ 4 mM CaCl_2_, pH 8.0) according to the size of pellet. The protein concentration was determined based on the Bradford method (Bradford Protein Assay, Bio-Rad).

100 μg protein from each sample was reduced by 10 mM DTT at 50 °C for 40 mins and then by the alkylation with 40 mM IAA (iodoacetamide) in the dark at room temperature for 30 mins. Subsequently, each sample was further diluted by distilled water for reducing urea concentration less than 2 M and sample peptides were acquired by a trypsin digestion (enzyme/protein, 1:50 w/w) at 37 °C overnight. The acquired acidified peptides by 10% trifluoroacetic acid (TFA) were further desalted by SepPak C18 cartridges (Waters). Moreover, the filtered peptide samples were speed-dried in a vacuum concentrator and directly dissolved into the water solution (0.1% formic acid) for MS analysis.

### SWATH-MS data analysis

MS analysis were performed by a Triple TOF 5600 mass spectrometer (AB Sciex) coupling with an Eksigent NanoLC-2D plus system. Peptide (2 μg) was trapped on a nanoFlexcHiPLC column (3 μm, ChromXP C18CL, 120 Å, 0.5 mm × 200 μm) under the 95% buffer A (water with 0.1% formic acid) and 5% buffer B (acetonitrile with 0.1% formic acid), followed by a separation through a 120-min gradient from 5 to 35% Buffer B at a stable flow rate of 300 nL/min. Full-scan MS was conducted in a positive ion mode with an ion spray voltage of 2300 V under the Triple TOF 5600 mass spectrometer. The reference spectral library was firstly constructed by the data-dependent acquisition (DDA) mode. The entire MS1 spectra was gathered by a full-scan of 250 ms from 350 to 1250 (m/z), among which the top 40 precursor ions were selected for MS/MS fragmentation (m/z 100–1800). In the SWATH acquisition mode, the similar chromatographic parameters was used as the DDA mode described above. A set of 50 overlapping windows was created to cover all precursor ions in the range of 400-1250 Da for further MS2 fragmentation through the cyclic DIA (Data-Independent Acquisition) mode. Thereafter, the DDA mass spectrometry files were searched by the software ProteinPilot 4.5 (AB Sciex) against from the Phytozme-*Malus domestica* v 1.0 protein database (63,517 protein entries, June 2019; https://phytozome.jgi.doe.gov/pz/portal.html). The parameters including the digestion, alkylation, biological modification and ID search were set up during the database search and a false discovery rate (FDR) of < 1% was applied into criteria selection for the protein identification and peptides assignments. Subsequently, all DIA raw files and reference library were loaded into the software PeakView v.1.2 (AB Sciex) using restricted parameters such as 8 peptides, 6 transitions, peptide confidence of > 99% and XIC width (50 ppm). The processed mrkvw files were followed by loading into MarkerView (AB Sciex) for further quantification analysis through the normalization of protein intensity of their peak areas as described in [58, 59]. Protein differential expression between Fuji_Apple and Fuji_M was expressed in ratios as > 1.5 or < 0.67 with *p* value < 0.05 to be up-regulation or down-regulation respectively. All the raw data files have been submitted into the PRIDE PRoteomicsIDEntifications (PRIDE) database with the accession number PXD011132.

### Proteomic data analysis

Gene Ontology (GO) analysis (http://geneontology.org/) and Kyoto Encyclopedia of Genes and Genomes (KEGG; http://www.kegg. jp/) enrichment classification were performed using DEPs data set.

## Supplementary information

**Additional file 1: Table S1.** Protein reports for the 1470 unique proteins quantified by SWATH-MS. (XLSX 538 kb)

**Additional file 2: Table S2.** List of differentially expressed proteins following classification analysis. Proteins with fold change > 1.5 (Increased) or < 0.67 (Decreased) are considered as DEPs (*P* value < 0.05). (XLSX 125 kb)

## Data Availability

The data sets are included within the article and its Additional files.

## Abbreviations

CHI: Chalcone isomerase
CHS: Chalcone synthase
DABR: Fruits were exposed to normal sunshine after bag removed
DAP: Days after pollination
DEPs: Differentially expressed proteins
DFR: Dihydroflavonol-4-reductase
DIA: Data-independent acquistion
F3H: Flavanone-3ß-hydroxylase
FDR: False discovery rate
FLS: Flavonol synthase
GO: Gene ontology
HSPs: Heat shock proteins
IAA: Iodoacetamide
LHC: Light-harvesting chlorophyll
MAPK4: Mitogen-activated protein kinase 4
MDH: Malate dehydrogenase
MEP: Methylerythritol 4-phosphate
MVA: Mevalonic acid
MVK: Mevalonate kinase
PSI: Photosystem I
SAMMTases: S-adenosyl-L-methionine-dependent methyltransferases
SRG: Splicing-related gene
SWATH-MS: Sequential window acquisition of all theoretical spectra-mass spectrometry
TFA: Trifluoroacetic acid
