# Supplementary figures and images for: SWATH-MS-facilitated proteomic profiling of fruit skin between Fuji apple and a red skin bud sport mutant
Source: BMC Plant Biol. 2019 Oct 24;19:445. doi: 10.1186/s12870-019-2018-1 (PMC6813987; doi:10.1186/s12870-019-2018-1)

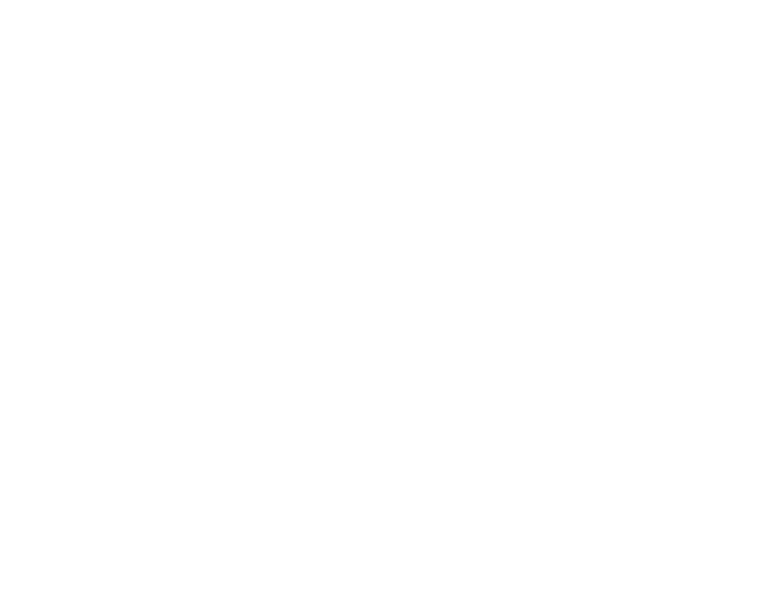

Supplement: Supplementary file 1 — Additional file 1: Table S1. Protein reports for the 1470 unique proteins quantified by SWATH-MS. (XLSX 538 kb) [file 12870_2019_2018_MOESM1_ESM.xlsx]

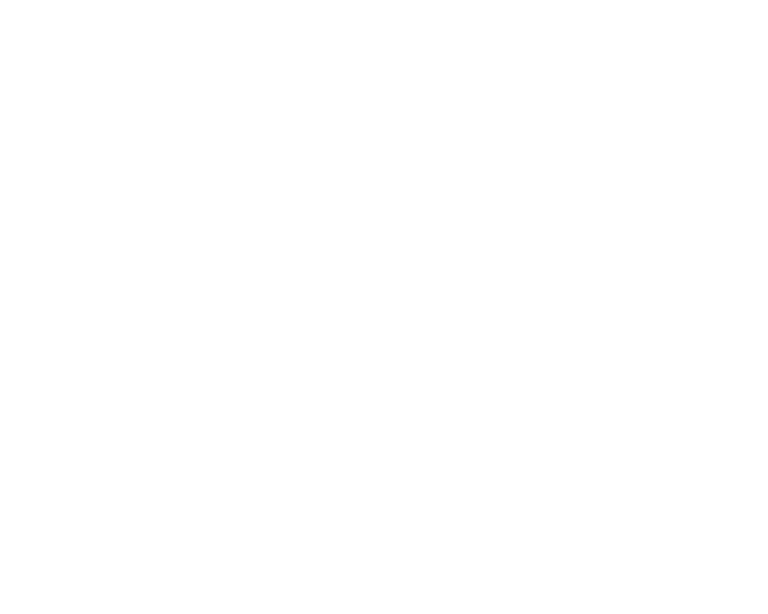

Supplement: Supplementary file 2 — Additional file 2: Table S2. List of differentially expressed proteins following classification analysis. Proteins with fold change > 1.5 (Increased) or < 0.67 (Decreased) are considered as DEPs (P value < 0.05). (XLSX 125 kb) [file 12870_2019_2018_MOESM2_ESM.xlsx]
